# The risk of revision following total hip arthroplasty in patients with inflammatory bowel disease, a registry based study

**DOI:** 10.1371/journal.pone.0257310

**Published:** 2021-11-04

**Authors:** Meghan M. Moran, Peter Wessman, Ola Rolfson, Daniel D. Bohl, Johan Kärrholm, Ali Keshavarzian, D. Rick Sumner

**Affiliations:** 1 Department of Cell & Molecular Medicine, Rush University Medical Center, Chicago, IL, United States of America; 2 Department of Orthopaedics, Institute of Clinical Sciences, Sahlgrenska Academy, University of Gothenburg, Gothenburg, Sweden; 3 Department of Orthopaedic Surgery, Rush University Medical Center, Chicago, IL, United States of America; 4 Division of Digestive Diseases and Nutrition, Department of Internal Medicine, Rush Medical College, Chicago, IL, United States of America; Center for Primary Care and Public Health, SWITZERLAND

## Abstract

Inflammatory bowel disease (IBD) is characterized by chronic inflammation of the intestinal tract and is associated with decreased bone mineral density. IBD patients are at higher risk of osteopenia, osteoporosis and fracture compared to non-IBD patients. The impact of IBD on the performance of orthopedic implants has not been well studied. We hypothesized that a history of IBD at the time of primary total hip arthroplasty (THA) would increase the risk of subsequent failure as assessed by revision surgery. A retrospective implant survival analysis was completed using the Swedish Hip Arthroplasty Registry and the Sweden National Patient Register. A total of 150,073 patients undergoing THA for osteoarthritis within an 18-year period were included in the study. THA patients with (n = 2,604) and without (n = 147,469) a history of IBD at the time of THA were compared with primary revision as the main endpoint and adjusted using sex, age category and comorbidity (Elixhauser scores) as covariates. We found that patients with a history of IBD had a relatively higher risk of revision surgery for septic causes while the non-IBD patients had a relatively higher risk of revision for aseptic causes (p = 0.004). Our findings suggest there may be an association between gut health and THA performance.

## Introduction

Inflammatory bowel disease (IBD) is a group of chronic inflammatory diseases that attack the intestinal tract, including Crohn’s disease (CD) and ulcerative colitis (UC) [1, 2]. IBD as a diagnosis is increasing world-wide [3]. IBD patients are most commonly diagnosed between 20 and 40 years of age [4], but a second peak of IBD diagnoses has presented in patients >60 years of age [4–6]. Older IBD patients account for ~10% of first-flare patients [7] and comprise 10–30% of the IBD population [8]. IBD-related intestinal symptoms can flare regularly and repeatedly throughout a patient’s life [4]. While CD can attack any region of the digestive track from the mouth to the anus, UC targets only the colon [9]. Location of disease within the intestinal track impacts local intestinal physiology including barrier function [10], and has downstream implications in remote organs including bone and joint in humans [11, 12] and in rats [13].

IBD patients are more susceptible to adverse effects in the skeleton compared to the non-IBD population. IBD patients have been shown to have low bone mineral density (BMD) due to increased bone resorption [14] and increased collagen breakdown [15], which is consistent with higher incidences of osteoporosis and fracture [1, 12, 16–21]. In fact, CD patients have a 60% higher risk of hip fracture [16, 22] and more generally, IBD patients have a 40% higher risk of other types of fractures, including spine and rib [23] compared to the general population. Further contributing to low BMD, an IBD diagnosis is associated with decreased bone formation serum markers (osteocalcin, bone alkaline phosphatase and C-terminal collagen propeptide) [20], decreased longitudinal bone growth in childhood and failure to attain normal peak bone mass [20, 24]. It has been shown that there is already significant reduction in trabecular bone by the time of CD diagnosis, suggesting bone is affected by IBD early in disease progression [25]. Drugs commonly prescribed for IBD have been shown to have mixed effects on the skeleton. Tumor necrosis factor-alpha (TNFα) targeting therapeutics lead to diminished bone quantity and quality [26] while corticosteroids have been associated with increased fracture risk in some [16] but not all [22, 27] studies.

Skeletal effects are exacerbated by CD-related nutritional deficiencies [21, 28, 29] and surgical and drug interventions. For example, ileal resection surgery can lead to malabsorption of vitamin D and calcium, which contributes to metabolic bone disease [30, 31]. IBD has also been associated with peripheral joint arthritis, which is present in 4.5% of IBD patients at the time of diagnosis and increases to 30% by the 20 year follow up [32]. Aging exacerbates these negative effects of IBD on the musculoskeletal system [27]. Thus, as patients with a history of IBD age, joint replacement surgery will likely become more prevalent in this population.

There has been limited study of orthopedic implant performance in IBD patients. Post-operative complications and revision rates following total hip arthroplasty (THA) are higher in some IBD patients [33]. Vitamin D deficiency, associated with CD, has also been linked to poor outcomes after total joint replacement [34, 35]. These studies indicate that there may be a link between IBD diagnosis and orthopedic implant outcomes.

The incidence of primary total hip and knee arthroplasty is expected to at least double in the next 30 years [36–40]. The impact of IBD on the lifespan of orthopedic implants is unknown. This study tested the hypothesis that the diagnosis of IBD is associated with decreased implant survivorship following THA.

## Materials and methods

### Ethical considerations

This study is part of a multidimensional research project based within the Swedish Hip Arthroplasty Register investigating factors associated with outcomes following hip replacement. Patients scheduled for THA are informed about the data collection in national registers in a written document that does not require a signature. Patients can, at any time, withdraw their consent actively, which means that all data about them, their surgery and follow-up information are deleted from the register. The ethical review board in Gothenburg approved the study and waived informed consent. The informed consent waiver is regulated within the Patient Data Act (2008:355) and the Act Concerning the Ethical Review of Research Involving Humans (2003:460).

### Study inclusion and exclusion criteria

**Fig 1:**
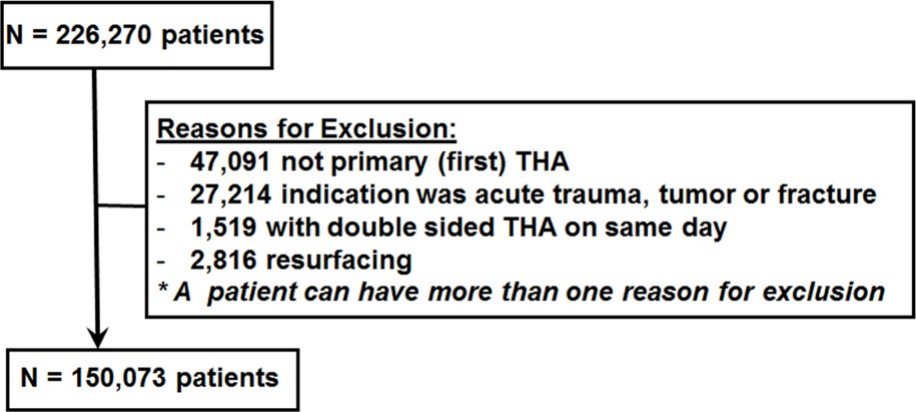
Flow diagram illustrating patient inclusion and exclusion criteria. The number of patients is described per inclusion and exclusion criteria throughout this figure. Patients were selected from the Swedish Hip Arthroplasty Register and the Sweden National Patient Register. THA- total hip arthroplasty.

We used the Swedish Hip Arthroplasty Registry and the Sweden National Patient Register to identify patients for this study, starting with 226,270 patients who had a primary THA within the 18 year period from January 1, 1999 to December 31, 2017 (Fig 1). Patients were excluded if they met one or more of the following criteria: 1) not the patient’s first THA (47,091), 2) THA for reasons other than primary OA (27,214), 3) double-sided THA (1,519), 4) patients who had resurfacing arthroplasties (2,816). Thus, a total of 150,073 patients who had a primary THA for osteoarthritis were included in the study (Fig 1).

### Classification of patients

The patients were identified as IBD (n = 2,604) based on having a history of ICD-10-SE (Swedish ICD-10) codes for CD (K50), UC (K51) or unspecified colitis (K52) at the time of their primary THA. For patient identification prior to 2015, ICD-9-SE codes were used CD (555), UC (556) or unspecified colitis (558). The remaining patients were classified as non-IBD (n = 147,469).

### Statistical analysis

The included patients were followed until December 31, 2017 (107,828) or death prior to December 31, 2017 (42,245). Data were summarized using absolute numbers and proportions for categorical variables and means and standard deviations (SD) for continuous variables. Pearson’s Chi-squared test was used to compare groups with regards to categorical variables. Time to all-cause mortality and time to revision were analysed using the Kaplan-Meier method. Cox proportional hazards models with IBD diagnose at index arthroplasty surgery were used to adjust for sex, age category, and comorbidity (Elixhauser scores) as covariates. Hazard ratios (HR) are presented with unadjusted 95% confidence intervals, Cox proportional hazard (PH) model assumption was assessed using stratified Kaplan-Meier and diagnostic plots. A competing risk analysis was performed for time to first revision with septic cause for revision, aseptic cause for revision and death as events. In addition, for all-cause mortality the relative survival compared to the general Swedish population was estimated, matched on gender and age. Relative survival is the ratio between observed survival and expected survival with a ratio over 1 indicating better survival than the general population and a ratio below 1 indicating excess mortality.

## Results

### Patient population

The prevalence of IBD at primary THA was slightly higher in females (1.9%) than males (1.5%, Table 1) with a prevalence ratio of 1.26 (95% CI: 1.16 to 1.36). The IBD and non-IBD patient’s mean age at the time of primary THA was 67.6 and 68.1 years, respectively. The categories of implant fixation included cemented, uncemented, hybrid (cemented fixation of the femoral component and cementless fixation of the acetabular component) and reverse hybrid (uncemented fixation of the femoral component and cemented fixation of the acetabular component). The type of fixation was different between the IBD and non-IBD groups (Table 1). Follow-up time (the time from THA to the last day of observation, 31 December 2017, or death) was slightly greater in the non-IBD group (8.9 years versus 7.3 years). The Elixhauser comorbidity index was higher in the IBD group than in the non-IBD group with 50.2% vs 36.5% of the patients, respectively, having a score above zero (p-value < 0.001).

**Table 1:** THA patient demographics by IBD diagnose at index THA surgery.

		Groups		
	Total	non-IBD	IBD	
**Total # of Patients**	150,073	147,469	2,604	
**# of Female (%)**	86,102 (57.4)	84,464 (57.3)	1,638 (62.9)	<0.001*
**# of Male (%)**	63,971 (42.6)	63,005 (42.7)	966 (37.1)	
**Age (yrs) (mean (SD))**	68.1 (10.6)	68.1 (10.63)	67.6 (11.26)	
**<55**	15,120 (10.1)	14,809 (10.0)	311 (11.9)	0.004*
**55–69**	63,128 (42.1)	62,028 (42.1)	1100 (42.2)	
**70–84**	65,830 (43.9)	64,750 (43.9)	1,080 (41.5)	
**85+**	5,995 (4,0)	5,882 (4.0)	113 (4.3)	
**Fixation (%)**				<0.001*
**Cemented**	115,056 (76.7)	113,152 (76.7)	1,904 (73.1)	
**Uncemented**	18,300 (12.2)	17,928 (12.2)	372 (14.3)	
**Hybrid**	3,874 (2,6)	3,808 (2.6)	66 (2.5)	
**Reverse hybrid**	12,824 (8.5)	12,562 (8.5)	262 (10.1)	
**Missing**	19	19	0	
**Follow-up time (yrs) (mean (SD))**	8.9 (4.4)	8.9 (4.4)	7.35 (3.70)	**<0.001**
**Elixhauser score (mean (SD))**	0.59 (0.96)	0.58 (0.95)	0.92 (1.22)	<0.001*
**Elixhauser index score (%)**				<0.001*
**0**	90,431 (63.2)	89,152 (63.5)	1,279 (49.8)	
**1**	31,802 (22.2)	31,134 (22.2)	668 (26.0)	
**2**	13,368 (9.3)	13,005 (9.3)	363 (14.1)	
**3**	4,993 (3.5)	4,849 (3.5)	144 (5.6)	
**4+**	2,414 (1,7)	2,300 (1.6)	114 (4.4)	
**Missing**	7,065	7,029	36	

**Fig 2:**
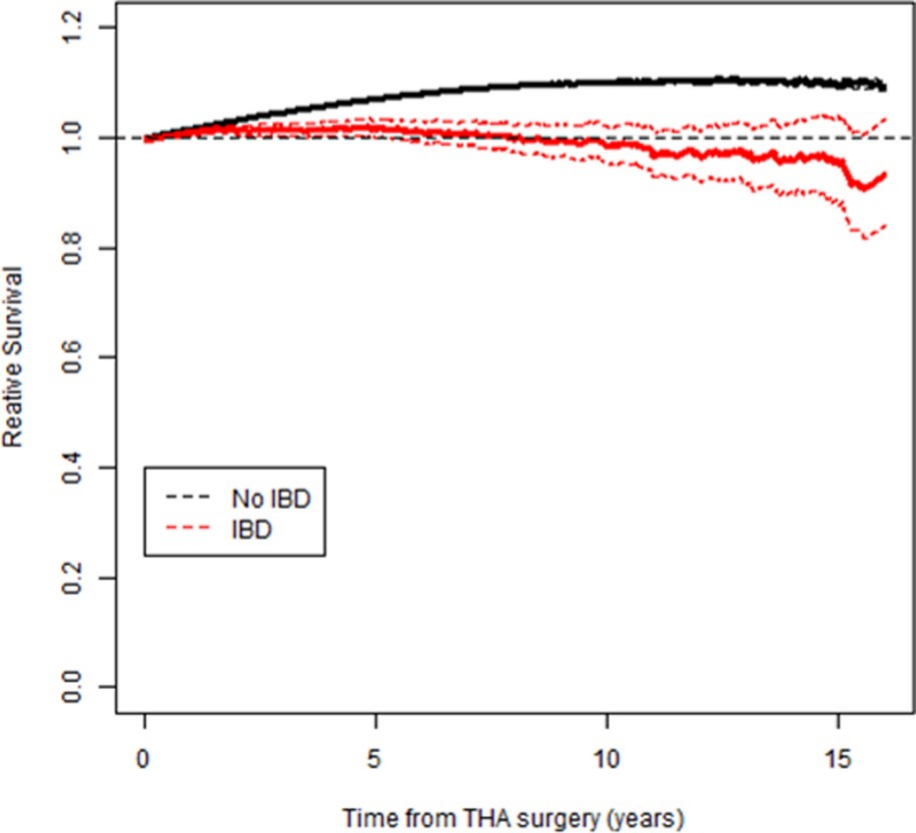
Relative survival of THA patients with and without IBD. With IBD, there is increased mortality compared to the general Swedish population. A curve above 1 indicates a lower risk for non-IBD THA patients compared to the general population. Dotted black line- general Swedish population. Solid black line- non-IBD, solid red line- IBD. Dotted black and red lines- 95% CI.

The relative survival of IBD THA patients was worse than the general Swedish population, while the relative survival of non-IBD THA patients was somewhat better than the general Swedish population (Fig 2).

**Fig 3:**
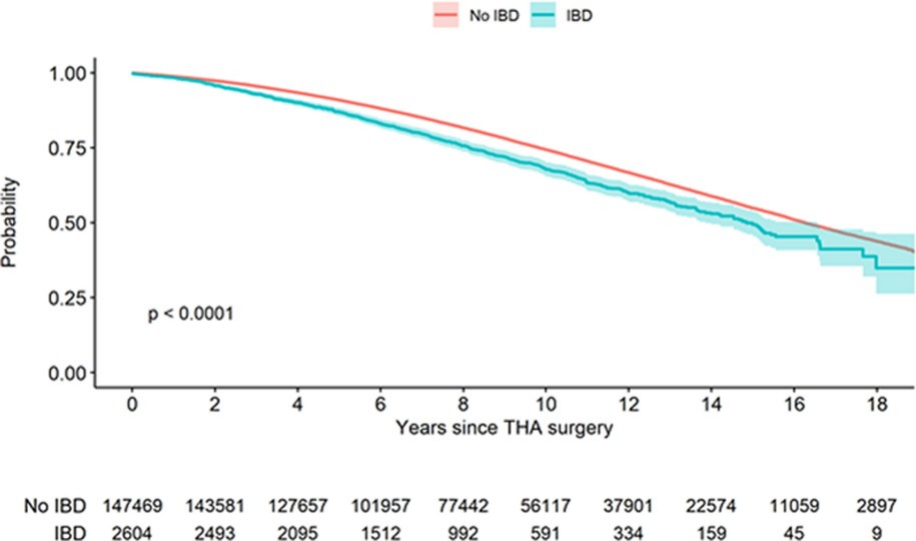
Patient mortality after primary THA. Kaplan-Meier curve stratified by IBD and non-IBD patients. THA patients with IBD have higher mortality compared to non-IBD THA patients (1.5 year shorter expected median survival). Solid aqua line- IBD, solid red line- non-IBD diagnosis, shaded areas- 95% CI.

Although the fraction of patients who died during follow-up did not differ among IBD (27.0%, 704/2,604) and non-IBD (28.2%, 41,560/147,469) groups, the median time to death was 1.5 years shorter for IBD patients, 14.8 years (95% CI: 13.9 to 16.6) compared to 16.3 years (95% CI: 16.2 to 16.4) (Fig 3, log-rank test: p<0.0001).

### Risk of revision

**Fig 4:**
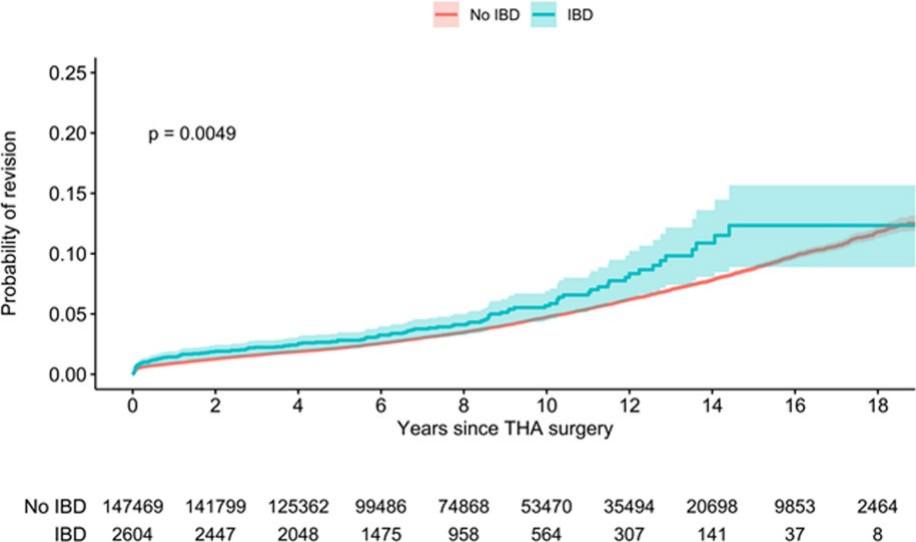
Kaplan Meier: Risk of revision after primary THA in patients with and without IBD at the time of index THA. Solid aqua line- IBD, solid red line- non-IBD diagnosis, shaded areas- 95% CI. Note that due to the sample size the red shaded area for the non-IBD CI is tightly fit to the red line.

IBD patients had a significant increased risk of revision compared to non-IBD patients (Fig 4, log-rank test, p-value = 0.0049). The probability of revision became significantly different 10 years after THA surgery. While the observed proportion of patients requiring revision during the follow-up period, which ranged from a minimum of 3 years to a maximum of 18 years, was not different between non-IBD and IBD patients (4.6% in both groups, Table 2), an adjusted Cox PH model including age, gender, comorbidity and IBD/non-IBD diagnosis showed an increased probability of revision for IBD compared to non-IBD patients (HR of 1.24, 95% CI: 1.03 to 1.48, S1 Table).

**Table 2:** Causes of revision.

	Groups		
	non-IBD	IBD	p-value
**Total # of Patients**	147,469	2,604	
**Patients requiring revision (%)**	6,754 (4.6)	120 (4.6)	0.945
**Aseptic v. Septic Cause (%)**			
**Peri-prosthetic infection (deep infection)**	3,112 (2.1)	71 (2.7)	0.004*
**Aseptic**	3,642 (2.5)	49 (1.9)	
**Aseptic Revision primary reason (%)**			0.924*
**Aseptic loosening**	3259 (89.4)	43 (87.8)	
**Peri-prosthetic Fracture**	366 (10.0)	6 (12.2)	
**Implant fracture**	13 (0.4)	0	
**Other aseptic reasons**	4 (0.1)	0	

In addition, there was a difference in the proportion of revisions due to septic and aseptic causes (p = 0.004). For septic cause, the observed risk of revisions was lower in non-IBD patients (2.1%) than in IBD patients (2.7%), (RR 0.77, 95% CI: 0.61 to 0.98). In contrast, the risk of revision due to aseptic causes was higher in non-IBD patients (2.5%) compared to IBD patients (1.9%), (RR 1.31, 95% CI: 0.99 to 1.74). The specific categories for aseptic revision were comparable between non-IBD and IBD patients (p = 0.924). The risk for revision due to septic cause, aseptic cause, or death was explored in a competing risk model (S1 Fig), which showed a higher risk of revision for sepsis in IBD patients compared to non-IBD patients (p = 0.02, S1 Table). The difference between IBD and non-IBD patients seen in the competing risk model seems to be driven largely by revisions due to septic causes.

## Discussion

With the occurrence of joint arthroplasty expected to increase steadily over the next 30 years, it is important to understand how bowel health impacts arthroplasty performance. Our study tested the hypothesis that there is an association between a diagnosis of IBD at the time of THA and decreased implant survivorship. We found that having IBD led to a greater risk of revision when age, gender and comorbidity index were included in the model. Specifically, there was a higher risk of revision for septic cause in IBD patients and a higher risk of revision for aseptic causes in non-IBD patients.

Our study highlighted the risk of septic complication of THA in patients with IBD and thus orthopedic surgeons should be aware of this risk and consider it when evaluating and discussing THA with IBD patients. Recently, increased effort has been applied to identifying risk factors for periprosthetic joint infection following joint replacement [41]. IBD could be a risk factor added to the list orthopedic surgeons use in risk stratification when considering whether surgery is worthwhile. Our study raises questions about whether additional steps could be taken to mitigate risk in IBD THA patients, including prolonged perioperative antibiotics, timing of surgery in relation with IBD disease activity or time of surgery in relation with time of IBD therapeutic administration or use of antibiotic cement. However, at this time, our study cannot provide any specifics on recommendations to mitigate increased risk. Our study does provided strong scientific rationale for future studies to determine the therapeutic approaches to mitigate this increase risk in IBD patients.

Our finding that IBD patients had a higher prevalence of sepsis as a cause for revision agrees with one study that found an increased risk of complications, including infection, post-THA surgery in IBD patients [33]. In contrast, a 2018 study found that IBD patients are not at increased risk of infection after orthopedic procedures [42]. Sepsis has been the second leading cause of implant failure, after aseptic loosening, for decades [43, 44]. Sepsis [45] and IBD [11, 12, 18, 20] have each been associated with decreased BMD. Although not measured in our study, low BMD, especially in the peri-implant region in the aged population, may contribute to the observed increase in risk of revision. CD-related low BMD is attributed to many factors including corticosteroid therapeutics, low vitamin D levels, being male and Asian ethnicity [46], amongst others. Corticosteroids, a commonly prescribed anti-inflammatory drug for IBD, have been shown to increase risk of osteoporotic fracture[22, 47] and vitamin D deficiency has been noted in both osteoporotic patients [48, 49] and IBD patients with decreased micronutrient absorption in the gut [34, 35, 49]. Together, these factors likely promote poor quantity and quality of peri-implant bone stock, contributing to implant failure.

Inflammation and the innate immune response are also altered in IBD patients [50] and may play a role in implant performance. In IBD patients, C-reactive protein (CRP), a systemic biomarker of inflammation, is significantly increased especially in CD [51] and pro-inflammatory macrophages and mast cells are found in higher concentrations in the intestine [50] compared to the general population. These inflammatory cells and associated inflammatory markers increase with age [52]. Age alone is a contributing factor in both IBD manifestation [5, 7] and THA survivorship and risk of revision [53]. Therefore, when all three factors present simultaneously, amplification of post-operative complications are likely to increase. While IBD is commonly thought of as a chronic disease of young adulthood, there is growing recognition of patients being diagnosed later in life, > 60 years of age [8]. The average age of patients in this study is ~68 years, with ~45% of the IBD patients being 70+ years old. The average age of patients undergoing primary arthroplasty is ~70 years [54] and revision surgeries occur later in life. These studies support that chronic inflammation, exacerbated by age, likely contribute to both gut health and arthroplasty outcomes.

Our findings suggest that gut health can play a role in THA outcomes. It is known that the intestinal microbiome of IBD patients is altered compared to non-IBD patients [55]. CD specifically is characterized by microbial dysbiosis and decreased diversity of the gut microbiome [9], even as early as the time of diagnosis prior to any therapeutic administration [55]. Alterations to the intestinal microbiome have also been shown to increase risk for systemic infection [56], affect bone mass [57, 58], and whole bone strength [59] in both humans and animal models. Disruption of the gut microbiome that results in abundance of pathogenic bacteria and decrease in short-chain fatty acid production are both factors in the risk and severity of sepsis [56]. Extensive rodent studies have shown that the gut milieu contributes to bone health [60]. Specifically, manipulation of the gut microbiota was shown to alter osteoarthritis [61, 62], rheumatoid arthritis [63, 64], fracture healing [65] and osteolysis [66]. Further studies using probiotic treatment showed increased bone density [67] and prevented bone loss following ovariectomy [68]. Taking into account the local and systemic effects IBD has on the body and the recognized importance of the gut-bone interaction, our results support that IBD is likely to affect arthroplasty outcomes.

In our study population, the prevalence of IBD was higher in females than males, which is consistent with previously published studies [69–71]. Effect modifiers that may have affected our results include sex and type of implant fixation. Sex may be an effect modifier because there are known disease differences between females and males. Type of implant fixation is known to influence implant survivorship [72, 73]. However, no major difference in revision among IBD and non-IBD patients was observed as a function of implant fixation type.

While, the relative survival of IBD THA patients was worse than the general Swedish population, non-IBD THA patients had better relative survival compared to the general Swedish population. This was likely because the non-IBD patients included in this study were healthy enough to benefit from THA.

Limitations of this study are inherent to registry studies, which include limited availability of data on potential confounding factors and under-reporting of outcomes if a patient leaves the registry or is not adequately followed up [74]. Our study also focuses only on patients that had primary surgeries within the 18 year time frame, and excludes patients with THA prior to January 1, 1999. There were a number of confounding factors that may be of interest for future studies, but which were not accessible in the data bases we used. The first is prescription drug use among our study population. IBD-associated medication including steroids, immune modulators (Azathioprine) and biologics (TNF antibody) increase risk of post-operative infection complication. Unfortunately, our database did not include a medication list and thus we cannot determine whether the increase risk of revision is due to medication or IBD. We were also unable to determine if IBD patients had an active or inactive disease state at the time of arthroplasty and the frequency and severity of active disease after their index THA. In addition, the lack of subgroup analysis of UC and CD is an important study limitation because their effects on the skeletal system may differ [16, 22, 23]. We explored stratifying the analyses by IBD disease subgroup, however, too much statistical power was lost, meaning that we may have masked important disease-specific effects.

## Conclusion

A history of IBD at the time of THA was associated with long-term greater risk of revision surgery due to sepsis.

## Supporting information

S1 Fig Probability of death and probability of revision due to aseptic and septic causes. Revisions due to septic cause occur earlier than revision due to aseptic cause regardless of IBD or non-IBD. Solid line- IBD, Dotted line- non-IBD, Black lines- death, red lines- septic cause for revision and blue lines- aseptic cause for revision.
(DOCX)

S1 Table Adjusted Cox PH model: Hazard ratio for time to first revision. Cox PH model including gender, age, comorbidity and IBD as main effects. No interaction effect was included.
(DOCX)
