# Supplementary material for: The risk of revision following total hip arthroplasty in patients with inflammatory bowel disease, a registry based study
Source: PLoS One. 2021 Nov 4;16(11):e0257310. doi: 10.1371/journal.pone.0257310 (PMC8568118; doi:10.1371/journal.pone.0257310)
Supplement: S1 Fig — Revisions due to septic cause occur earlier than revision due to aseptic cause regardless of IBD or non-IBD. Solid line- IBD, Dotted line- non-IBD, Black lines- death, red lines- septic cause for revision and blue lines- aseptic cause for revision. (DOCX) [file pone.0257310.s001.docx]

**
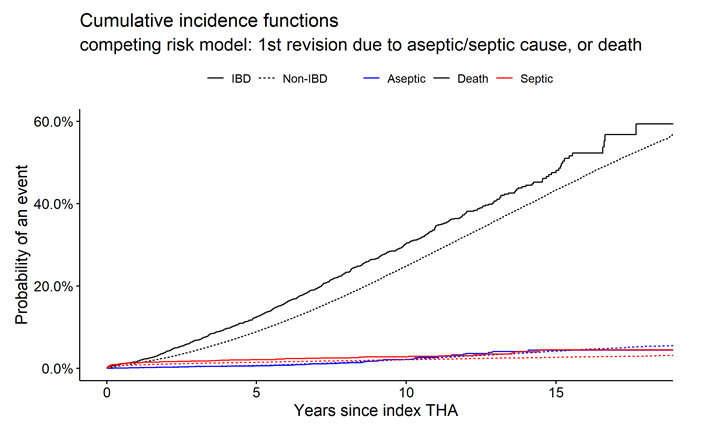
**

**Figure S1. Probability of death and probability of revision due to aseptic and septic causes.** Revisions due to septic cause occur earlier than revision due to aseptic cause regardless of IBD or non-IBD. Solid line- IBD, Dotted line- non-IBD, Black lines- death, red lines- septic cause for revision and blue lines- aseptic cause for revision.
