# Supplementary material for: The risk of revision following total hip arthroplasty in patients with inflammatory bowel disease, a registry based study
Source: PLoS One. 2021 Nov 4;16(11):e0257310. doi: 10.1371/journal.pone.0257310 (PMC8568118; doi:10.1371/journal.pone.0257310)
Supplement: S1 Table — Cox PH model including gender, age, comorbidity and IBD as main effects. No interaction effect was included. (DOCX) [file pone.0257310.s002.docx]

|  | **Levels** | **All** | **HR (univariable)** | **HR (multivariable)** |
| --- | --- | --- | --- | --- |
| **Gender** | Female | 86102 (100.0) |  |  |
|  | Male | 63971 (100.0) | 1.42 (1.36-1.48, p < 0.001) | 1.36 (1.30-1.43, p < 0.001) |
| **Age** | <55 | 15100 (100.0) |  |  |
|  | 55-69 | 63128 (100.0) | 0.78 (0.73-0.83, p < 0.001) | 0.78 (0.73-0.83, p < 0.001) |
|  | 70-84 | 65830 (100.0) | 0.53 (0.49-0.57, p < 0.001) | 0.54 (0.50-0.58, p < 0.001) |
|  | 85+ | 5995 (100.0) | 0.54 (0.45-0.64, p < 0.001) | 0.55 (0.46-0.66, p < 0.001) |
| **Elixhauser** | 0 | 90431 (100.0) |  |  |
|  | 1 | 31802 (100.0) | 1.11 (1.05-1.18, p = 0.001) | 1.15 (1.09-1.23, p < 0.001) |
|  | 2 | 13368 (100.0) | 1.15 (1.05-1.26, p = 0.003) | 1.23 (1.12-1.35, p < 0.001) |
|  | 3 | 4993 (100.0) | 1.17 (1.00-1.36, p = 0.046) | 1.28 (1.10-1.49, p < 0.001) |
|  | 4+ | 2414 (100.0) | 1.75 (1.45-2.12, p < 0.001) | 1.91 (1.57-2.31, p < 0.001) |
|  | Missing | 7065 (100.0) | 0.96 (0.87-1.06, p = 0.457) | 0.95 (0.86-1.05, p < 0.001) |
| **IBD** | Non-IBD | 147469 (100.0) |  |  |
|  | IBD | 2604 (100.0) | 1.30 (1.08-1.55, p = 0.005) | 1.24 (1.03-1.48, p = 0.020) |
| *Model metrics:* Number in dataframe= 150073. Number in model = 150073. Missing = 0. Number of events = 6874. Concordance = 0.574 (SE = 0.004). R-squared = 0.004 (max possible= 0.664). Likelihood ratio test = 593.995 (df = 10, p = 0.000) | | | | |
|  |  |  |  |  |

**Supplemental Table 1.** Adjusted Cox PH model: Hazard ratio for Time to first Revision. Cox PH model including gender, age, comorbidity and IBD as main effects. No interaction effect was included.
